# The Effects of Sex and Age on Sympathetic Neurovascular Control of Cerebral Hemodynamics

**DOI:** 10.1002/alz70855_101079

**Published:** 2025-12-23

**Authors:** Yasmine Coovadia, Kathleen B. Miller, Nicole A. Loggie, Adam T. Corkery, Anna J. Howery, Jill N. Barnes

**Affiliations:** ^1^ University of Wisconsin‐Madison, Madison, WI, USA; ^2^ University of St. Thomas, Saint Paul, MN, USA

## Abstract

**Background:**

Blood pressure (BP) dysregulation can significantly disrupt brain circulation, which in turn increases the risk of developing Alzheimer's disease. Both cerebral blood flow (CBF) and BP regulation are influenced by biological sex and aging. Notably, females experience a more pronounced age‐related decline in CBF and more substantial changes in autonomic BP regulation compared with males, which may contribute to their heightened risk for Alzheimer's disease. However, the specific impact of aging and biological sex on the relationship between autonomic BP fluctuations and CBF is poorly understood. As such, this study explored the hypothesis that older females experience more significant impairments in sympathetic BP regulation and CBF compared to both older males and younger individuals.

**Method:**

We recruited healthy participants across two age groups: young (18‐35 years) males (*n* = 25) and females (*n* = 17), and older (55‐69 years) males (*n* = 14) and females (*n* = 15). After instrumentation we measured middle cerebral artery velocity (MCAv) as an indicator of CBF using Doppler ultrasound, BP (mean arterial pressure) via finger photoplethysmography, and integrated sympathetic nerve activity (SNA; quantified as “bursts” of activity) using peroneal microneurography. Fluctuations in BP and MCAv were tracked on a beat‐by‐beat basis following each heartbeat in which an SNA burst was identified.

**Result:**

Increases in BP following SNA bursts were significantly attenuated in older participants compared to younger participants (*p* <0.05), though no differences were found between sexes (*p* = 0.74). Similarly, increases in MCAv following SNA bursts were reduced in older participants relative to younger participants (*p* <0.05); however, MCAv increases following SNA bursts were lower in both groups of females compared with both groups of males (*p* <0.05). Furthermore, peak increases in BP following a burst were positively correlated with peak increases in MCAv in young participants (males: R=0.40, *p* <0.05; females: R=0.52, *p* <0.05) and older males (R=0.72, *p* <0.05), whereas there was no correlation observed in older females (R=0.32, *p* = 0.14).

**Conclusion:**

Collectively, these findings suggest that older females experience a disconnect between sympathetic BP regulation and CBF fluctuations. This is in contrast to young healthy adults and older males. This mechanism may contribute to the increased risk of cognitive impairments in aging females.

